# Induction of long-lived room temperature phosphorescence of carbon dots by water in hydrogen-bonded matrices

**DOI:** 10.1038/s41467-018-03144-9

**Published:** 2018-02-21

**Authors:** Qijun Li, Ming Zhou, Mingyang Yang, Qingfeng Yang, Zhixun Zhang, Jing Shi

**Affiliations:** State Key Laboratory of Tribology, School of Mechanical Engineering, Tsinghua University, Beijing, 100084 China

## Abstract

Phosphorescence shows great potential for application in bioimaging and ion detection because of its long-lived luminescence and high signal-to-noise ratio, but establishing phosphorescence emission in aqueous environments remains a challenge. Herein, we present a general design strategy that effectively promotes phosphorescence by utilising water molecules to construct hydrogen-bonded networks between carbon dots (CDs) and cyanuric acid (CA). Interestingly, water molecules not only cause no phosphorescence quenching but also greatly enhance the phosphorescence emission. This enhancement behaviour can be explained by the fact that the highly ordered bound water on the CA particle surface can construct robust bridge-like hydrogen-bonded networks between the CDs and CA, which not only effectively rigidifies the C=O bonds of the CDs but also greatly enhances the rigidity of the entire system. In addition, the CD-CA suspension exhibits a high phosphorescence lifetime (687 ms) and is successfully applied in ion detection based on its visible phosphorescence.

## Abstract (web summary)

A long phosphorescence lifetime is desirable for imaging and detection, however, phosphorescence is often quenched in aqueous environments limiting applications. Here, Li et al. present a strategy for the long-lived phosphorescence of carbon dots in water due to multiple hydrogen-bonding interactions

## Introduction

Room temperature phosphorescence (RTP) has attracted growing attention because of its long-lived luminescence, large stokes shift and high signal-to-noise ratio, allowing its application in the fields of optoelectronic devices^1–4^, bioimaging^5–7^, chemical sensors^8–10^ and so forth. Most current RTP materials show phosphorescent features in their anhydrous solid states^11–13^, but phosphorescence quenching usually occurs in the presence of water owing to the presence of dissolved oxygen as well as the occurrence of solvent-assisted relaxation^14^, impeding the aqueous applications of RTP materials. Though few species with phosphorescence in aqueous media have been reported^15,16^, most of them exhibit relatively short emission lifetimes. In addition, the present RTP materials are mainly organometallic^4,17,18^ and crystalline organic compounds^19–21^. However, all of these materials suffer from high costs, cytotoxicity and complicated preparation processes. Therefore, the development of a facile strategy for preparing efficient RTP materials in aqueous environments is desirable.

Recently, carbon dots (CDs) have aroused increasing attention as a new type of photoluminescent (PL) nanomaterial because of their high photostability, low toxicity and small size^22–24^. Generally, CDs show strong fluorescence, but minimal phosphorescence is observed at room temperature^25–28^. However, when CDs are fixed in a certain matrix, such as poly(vinyl alcohol)^29–32^, KAl(SO_4_)_2_·x(H_2_O)^33^ or polyurethane^34^, obvious phosphorescence can be detected under excitation by ultraviolet light. Most recently, in 2016, an ultralong phosphorescence lifetime (up to 1.06 s) was achieved in our laboratory from CDs and composite matrices^35^. The matrix can effectively protect the CD triplet states from quenching by restricting their intermolecular motions. However, nearly all RTP materials based on CDs emit phosphorescence only in their dry state. Phosphorescence would undergo extensive quenching in the presence of water because water molecules usually break up hydrogen-bonding interactions between CDs and matrices. In addition, the dissolved oxygen present in water can further contribute to phosphorescence quenching. These unfavourable factors severely hinder the aqueous application of RTP materials, particularly in the fields of bioimaging and chemical sensors.

The water that exists in the vicinity of the matrix can generally be divided into three types: bulk water, freezing bound water, and non-freezing bound water. Bound water combines with proteins^36,37^, phospholipids^38^ and other solid substances,^39^ mainly through hydrogen bonds. Compared with bulk water, bound water exhibits unique structural properties, including difficulty freezing, difficult separation, and restricted mobility and solubility, which endow it with unique applications^36,40,41^. A recent study^42^ indicated that the bound water confined in a polyelectrolyte brush exhibited a tight arrangement with a high density, which greatly enhanced the hydrogen-bonded network. The strong hydrogen-bonding structures of water in swollen polymer brushes have been reported to suppress protein adsorption, affording biocompatibility^43^. Based on these conclusions, it is speculated that if we utilise bound water to construct hydrogen-bonded networks between CDs and matrices, effective phosphorescence may be achieved, even in aqueous environments. The achievement of such phosphorescence emission by utilising bound water is considered to be important in biological applications, such as cell imaging and disease diagnosis, as bound water widely exists in cells and biological tissues.

Herein, a facile strategy for preparing a long-afterglow RTP material by utilising water molecules to construct hydrogen-bonded networks between CDs and cyanuric acid (CA) particles is reported. Our results show that water molecules not only cause no phosphorescence quenching but also greatly enhance the phosphorescence emission. This abnormal enhancement behaviour may be attributed to the water-induced formation of hydrogen-bonded networks between CDs and CA particles. In the presence of water, the surfaces of the CA particles can strongly absorb a layer of highly ordered water molecules (non-freezing bound water). These bound water molecules can construct robust bridge-like hydrogen-bonded networks between the CDs and CA particles, which not only effectively rigidifies the C=O bonds of CDs but also greatly enhances the rigidity of the entire system, resulting in the enhancement of phosphorescence. In addition, the CD-CA suspension exhibits a phosphorescence lifetime of 687 ms under 373 nm excitation and is successfully applied in ion detection based on its visible phosphorescence. As an example, the detection limit of Fe^3+^ ions is as low as 32 µM. The CD-CA suspension can also be successfully applied to the determination of Fe^3+^ in real lake water and complicated protein solutions.

## Results

### Characterisation of the CDs and CD-CA powder

**Fig. 1:**
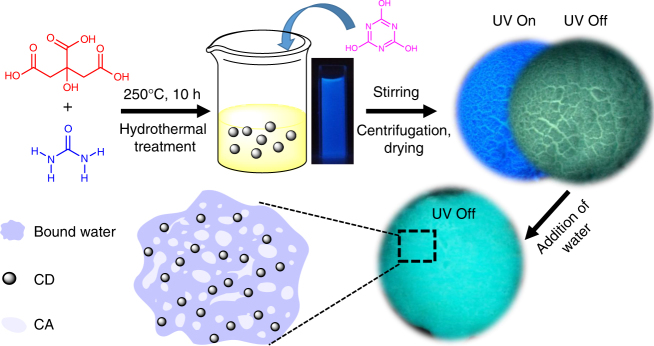
Preparation of the CDs and the CD-CA system. Schematic illustration for the preparation of the CDs and the CD-CA system

**Fig. 2:**
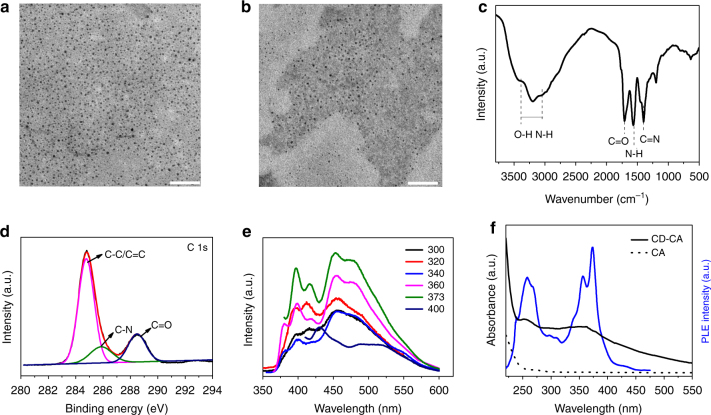
Characterisation of the CDs and CD-CA powder. **a** TEM image of the CDs (scale bar, 50 nm). **b** TEM image of the CD-CA nanohybrid (scale bar, 50 nm). **c** FTIR spectrum of the CDs. **d** High-resolution C 1 s XPS spectrum of the CDs. **e** Phosphorescence spectra of the CD-CA powder under different excitation wavelengths. **f** Phosphorescence excitation spectrum (blue solid line) of the CD-CA powder with emission at 480 nm and the absorption spectra of the CD-CA power (black solid line) and the CA powder (black dotted line)

The preparation of CDs and the CD-CA powder is described in Fig. 1. The CD aqueous solution and CD-CA powder emitted blue light under illumination by UV (365 nm) light. Interestingly, when the ultraviolet source was removed from the CD-CA powder, a green afterglow was observed with the naked eye at room temperature. The morphologies and optical properties of the CDs in solution and the CD-CA powder were studied. As shown in Fig. 2a, a transmission electron microscopy (TEM) image of the CDs demonstrates the formation of well-dispersed nanoparticles with a mean particle diameter of 4.8 nm (Supplementary Fig. 1a). An atomic force microscopy image of the CDs reveals that their topographic heights are in the range of 0.5–2.5 nm, suggesting that the prepared CDs are anisotropic (Supplementary Fig. 1b). The related high-resolution TEM (HRTEM) image and X-ray diffraction (XRD) results reveal the presence of both crystalline graphite and amorphous phases (Supplementary Fig. 1c, d). In the presence of CA, the CDs evidently cover the surface of CA in their assembled state, and the morphology of the CDs mediated by CA exhibits no significant changes relative to that of the original CDs (Fig. 2b). The Fourier transform infrared (FTIR) spectrum of the CDs (Fig. 2c) displays characteristic peaks at 1705, 1565 and 1400 cm^−1^ that are attributed to C=O, N–H, and C–N stretching vibrations, respectively. The results of X-ray photoelectron spectroscopy (XPS) experiments indicate that the CDs mainly consist of carbon, nitrogen, and oxygen (Supplementary Fig. 1e). The C 1 s XPS spectrum (Fig. 2d) shows three peaks at 284.6, 285.6 and 288.1 eV for C–C, C–N and C=O, respectively^44^. Fig 2e shows the corresponding phosphorescence spectra of the CD-CA powder under different excitation wavelengths, where an optimal excitation at 373 nm was obtained. To investigate the origin of the observed phosphorescence, the phosphorescence excitation and absorption spectra of the CD-CA powder were investigated (Fig. 2f). In the absorption spectrum, two bands centred at 255 and 360 nm were observed, which are owing to the π–π* transition of the aromatic *sp*^*2*^ domains and the n–π* transition of the C=O bonds, respectively^45^. The phosphorescence emission of the CD-CA powder at 480 nm could be excited within the range of 300–400 nm, which overlaps with the absorption bands of C=O, suggesting that the observed phosphorescence may come from the surface C=O bonds of the CDs.

### The role of water in phosphorescence

**Fig. 3:**
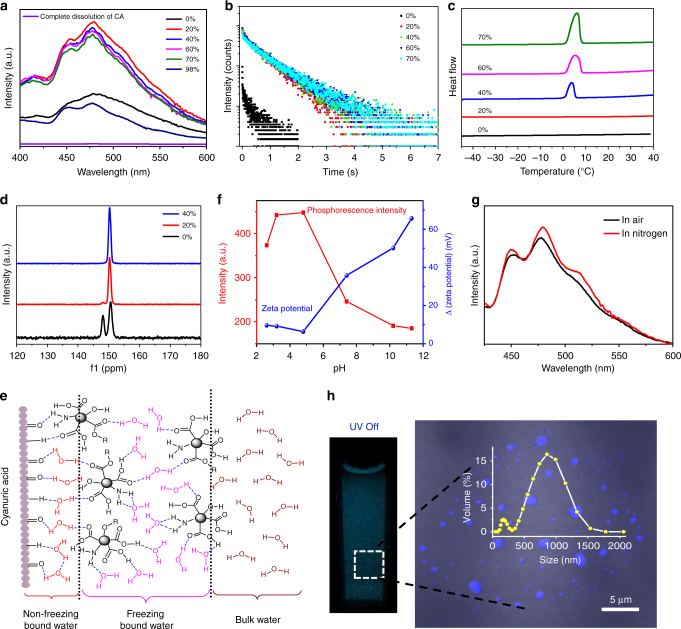
Role of water in phosphorescence. **a** Phosphorescence spectra and **b** lifetime decay profiles of the CD-CA system with various water contents under excitation at 373 nm. **c** Representative DSC-heating curves of the CD-CA system with various water contents as a function of temperature. **d** ^13^C NMR spectra of the CD-CA system with various water contents. **e** Schematic illustration of the molecular interactions between the CDs, CA particles and water molecules. **f** Phosphorescence intensity at 480 nm of the CD-CA suspension as a function of the pH (red line) and the sum of the absolute values of the CD and CA zeta potentials as a function of pH (blue line). **g** Phosphorescence spectra of the CD-CA suspension in air-saturated and nitrogen (N_2_) conditions under excitation at 373 nm. **h** Image (left) of the CD-CA suspension treated by ultrasound and low-speed centrifugation after the UV light was turned off; corresponding fluorescence image (right), as determined by CLSM; corresponding size distribution (inset), as determined by DLS

The phosphorescence of the CD-CA powder could be significantly enhanced by adding a certain amount of water. Notably, the intensity of phosphorescence in the presence of water was nearly three times higher than that of the dry CD-CA powder, and the average lifetime increased from 0.253 s for the dry CD-CA powder to 0.687 s for the water-added CD-CA powder (Fig. 3a, b), whereas this enhancement was not observed with other common organic solvents (e.g., ethanol, methanol, acetone, and tetrahydrofuran) (Supplementary Fig. 2a). This interesting water-triggered phosphorescence feature endows the CDs with great potential for application in optical recording media and double anti-counterfeiting (Supplementary Fig. 2b and Supplementary Note 1). Water molecules are known to usually break up intermolecular hydrogen-bonding interactions, resulting in a significant decrease in phosphorescence. However, the distinctive enhancement property observed here may be attributed to the formation of unique intermolecular interactions among the CDs, CA particles and water molecules. First, the possibility that the phosphorescence enhancement was caused by a change in the concentration before and after the addition of water was ruled out because the absorption spectra of the material in the absence and presence of water are almost identical (Supplementary Fig. 2c). Moreover, nearly identical PL decay curves were obtained before and after the addition of water, further excluding the self-quenching of the CDs in the dry state (Supplementary Fig. 2c). In addition, the zeta potential results indicate that both the CDs and CA particles have negative potentials in aqueous solution (Supplementary Table 1), indicating the presence of repulsive electrostatic forces between the CDs and CA particles; thus, the possibility that the phosphorescence enhancement in this experiment was induced by electrostatic attraction is also unlikely. When we tried to enhance the phosphorescence of the CD-CA powder by adding plenty of water, we found that the phosphorescence enhancement was not linearly related to the water content. As shown in Fig. 3a, an obvious enhancement in the phosphorescence was observed for a 20% water content, and the intensity of the phosphorescence remained nearly unchanged with a further increase in the water content up to 70%, but when the CD-CA powder dissolved after adding a large amount of water (98%), the phosphorescence decreased and disappeared completely in the dissolved state of the CD-CA powder. To explain the mechanism, we investigated the different states of water present in the CD-CA system by differential scanning calorimetry (DSC) (Fig. 3c). An endothermic melting peak was not observed at a low water content (20%), suggesting that this portion of water is of the non-freezing bound type. As the content of water gradually increased from 20% to 70%, a broad endothermic peak appeared and moved to higher temperatures, which indicates the presence of two states of freezing water (freezing bound water and bulk water) in the composite. More robust exothermic signals were observed at high water contents, corresponding to an increase in the bulk water content. Although the quantification of each state of water by DSC is difficult due to the overlap between the two melting peaks, we can confirm that all the water in the sample with a 20% water content is non-freezing bound water. Based on the results presented above, the observed phosphorescence enhancement is mainly attributed to non-freezing bound water, while bulk water had a negligible effect on the phosphorescence. A possible mechanism for the water-enhanced phosphorescence emission is illustrated in Fig. 3e. CA is a unique cyclic amide consisting of three hydrogen bond donors and three acceptor sites, leading to a range of hydrogen-bonding possibilities. In the presence of water, the CA particle surfaces can strongly absorb a layer of highly ordered water molecules (non-freezing bound water). This type of water exhibits unique structural properties, including difficulty freezing, difficult separation, and restricted mobility and solubility, and constructs robust bridge-like hydrogen-bonded networks between the CDs and CA particles, which not only effectively rigidifies the C=O bonds of CDs but also greatly enhances the rigidity of the entire system, resulting in the enhancement of phosphorescence. To further demonstrate the bridging role of the water molecules, ^13^C-NMR spectra of the CD-CA system were measured (Fig. 3d). In the dry state, the ^13^C-NMR spectrum of CD-CA contains two distinct, non-equivalent peaks at 148.1 and 150.6 ppm that arise from the different hydrogen-bonding environments in the supramolecular assembly (the hydrogen bonds formed by CA with itself through different sites and those formed between CA and CDs). With an increase in the water content from 0 to 40%, the peak at 148.1 ppm becomes weaker and shifts to 150.2 ppm. Such a phenomenon can be explained by the fact that water molecules can construct robust bridge-like hydrogen-bonded networks between the CDs and CA particles and replace the hydrogen bonds formed by CA with itself or between CA and CDs, resulting in the disappearance of the asymmetric hydrogen-bonding interactions that are present in the CD-CA dry state. In addition, the Raman spectra of CA further demonstrated the presence of hydrogen-bonding interactions between water molecules and the CA particles (Supplementary Fig. 2d and Supplementary Note 1). Compared with bound water, bulk water is not intimately bound to the CA particle surface but rather interacts weakly with the CA particles, leading to a negligible effect on phosphorescence. However, when dissolution of the CD-CA powder occurred, the number of water molecules bound to the CA particles began to decrease until they were all converted to bulk water in the completely dissolved state. At this point, the hydrogen-bonded network between the CA particles and CDs collapsed, resulting in the disappearance of phosphorescence.

To obtain better insight into the bonding interactions between the CDs and CA molecules in the completely dissolved state of the CD-CA powder, their thermodynamic parameters and binding constants were measured by isothermal titration calorimetry (ITC) and microscale thermophoresis (MST) (Supplementary Fig. 3a–d and Supplementary Note 1). The obtained endothermic signal and positive entropy values suggest that the binding of CDs to CA molecules is partly driven by hydrophobic interactions, which may be attributed to the hydrophobic carbon skeleton of the CDs. A low binding constant indicates the presence of weak binding interactions between the CDs and CA, explaining the absence of phosphorescence in the completely dissolved state of the CD-CA powder.

The optical spectra of CD-CA powders with various CD contents were recorded (Supplementary Fig. 4a and Table 2 and Supplementary Note 1). At low CD concentrations, the phosphorescence lifetime and fluorescence quantum yield remained nearly unchanged, whereas both properties showed obvious quenching at high concentrations, which is owing to an increase in the level of self-absorption by the CDs. In addition, we investigated the effects of the aqueous environment pH on phosphorescence . The emission intensity of the CD-CA system showed a negligible change over the pH range from 3.1 to 4.8 and a sudden reduction at pH 7.4. To explain this phenomenon, the zeta potentials of the CDs and CA particles were measured. The sum of the absolute values of the CD and CA particle zeta potentials exhibited an opposite trend as that of the emission intensities of the CD-CA system at different pH values (Fig. 3f and Supplementary Table 1), suggesting that electrostatic interactions have an important role in phosphorescence. In addition, an obvious reduction in the amount of bound water in the CD-CA system at pH 7.4 compared with that at pH 4.8, which causes a further reduction in the phosphorescence of the CD-CA system at pH 7.4, was detected by DSC tests (Supplementary Fig. 4b). Dissolved oxygen is known to often cause phosphorescence quenching in water. Thus, the phosphorescence spectra of CD-CA suspensions with and without nitrogen bubbling were measured, and almost identical optical curves were obtained (Fig. 3g), which may be attributed to the restricted solubility of oxygen in bound water. In a further set of experiments, we investigated the effects of the temperature on the photoluminescence of the CD-CA system containing 40% water in the range of 78–301 K. (Supplementary Fig. 4c, d, Table 3 and Supplementary Note 1). Usually, the phosphorescence intensity increases as the temperature decreases^30,46^; however, our results were different. As shown in Supplementary Fig. 3c, the phosphorescence of the CD-CA system decreased with a decrease in the temperature, which might be attributed to the freezing water in the CD-CA system. Small phosphorescent materials are very important in biology and related fields. The phosphorescence image, confocal laser scanning microscopy (CLSM) results and dynamic light scattering (DLS) data (Fig. 3h) illustrated that the CD-CA suspension still exhibited visible phosphorescence under illumination by UV light at the nanometre scale, which is crucial for real applications.

### Application in ion detection

**Fig. 4:**
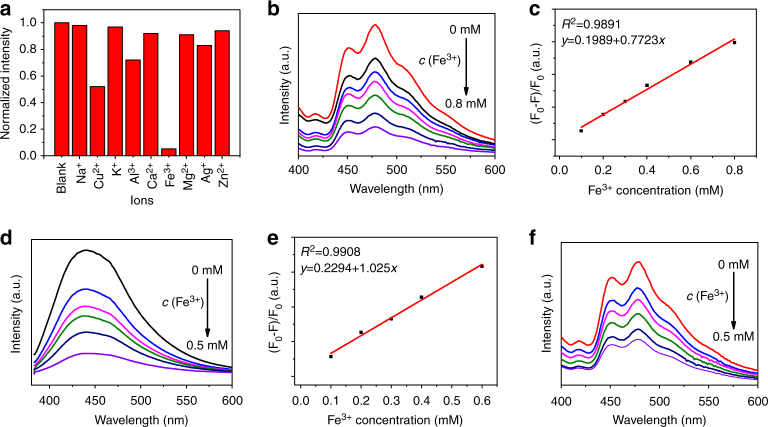
Detection of Fe^3+^ ions. **a** Normalised phosphorescence intensity of the CD-CA suspension in the presence of various metal ions. **b** Phosphorescence spectra of CD-CA suspensions with various calculated concentrations of Fe^3+^; from top to bottom: 0, 0.1, 0.2, 0.3, 0.4, 0.5 and 0.8 mM. **c** Stern–Volmer plot as a function of Fe^3+^ concentration. **d** Fluorescence spectra of CDs at various calculated concentrations of Fe^3+^; from top to bottom: 0, 0.1, 0.2, 0.3, 0.4 and 0.5 mM. **e** Stern–Volmer plot as a function of Fe^3+^ concentration. **f** Phosphorescence spectra of CD-CA suspensions at various calculated concentrations of Fe^3+^ in a peptone solution; from top to bottom: 0, 0.1, 0.2, 0.3, 0.4 and 0.5 mM

Ion detection based on CD fluorescence has recently undergone rapid development^23,47^, but this application can be conducted in only non-fluorescent aqueous solutions^48,49^. Many proteins in living organisms and algae in polluted water have strong fluorescence emissions, leading to the failure of the fluorescent probe. Phosphorescent detection avoids interference from the short-lifetime fluorescent background or scattered light in real samples^50^, providing a higher sensitivity and signal-to-noise ratio of detection in complicated media. Taking advantage of the long-lived persistent RTP in aqueous media, the CD-CA suspension could be utilised as a sensor for the detection of metal ions in water. The test results indicate that the phosphorescence of the CD-CA suspension can be selectively quenched in the presence of Fe^3+^ (Fig. 4a). The phosphorescence intensity gradually decreased as the concentration of Fe^3+^ ions increased (Fig. 4b). A good linear relationship within the range from 0.1 to 0.8 mM with a correlation coefficient (*R*^2^) of 0.9891 was obtained (Fig. 4c). The limit of detection was estimated to be 32 μM based on the rule of three times the standard deviation. We also investigated the phosphorescence quenching effect of other metal ions on the CD-CA suspension. As shown in Fig. 4a, Fe^3+^ ions had the strongest ability to quench the phosphorescence, while other ions showed a negligible quenching effect. In addition, we found that the fluorescence of the CDs could also be selectively be quenched by Fe^3+^ (Fig. 4d, e). The simultaneous fluorescence and phosphorescence quenching by Fe^3+^ was not a coincidence. The quenching effect presumably results from static quenching arising from the formation of a non-fluorescent complex between the CDs and Fe^3+^. This non-luminescent chelate changes the original CD energy level structure created by the surface carbonyl or hydroxyl groups. Combining the above analysis, the C=O bonds on the CD surface are mainly responsible for the observed phosphorescence. Thus, the change in the energy level structure leads to phosphorescence quenching. To gain further insight into the PL quenching mechanism, the fluorescence lifetime, phosphorescence lifetime and UV-vis absorption spectra were obtained (Supplementary Fig. 5a–c). The fluorescence lifetime of the CDs remained nearly constant before and after the coordination of Fe^3+^ ions, as did the phosphorescence lifetime of the CD-CA suspension. Moreover, the absorption peaks of the CDs showed obvious differences in the absence and presence of Fe^3+^. These results clearly indicate that the quenching mechanism of the CDs by Fe^3+^ occurs through a static quenching process.

In further experiments, we applied the CD-CA probe to Fe^3+^ determination in complicated peptone solutions containing numerous amino acids and proteins. As anticipated, the phosphorescent sensor still showed a good linear correlation in the concentration range from 0.1−0.5 mM with a detection limit of 28 µM, but the fluorescent sensor based on CDs did not work well because of interference by scattering light and fluorescent organics (Fig. 4f and Supplementary Fig. 5d, e). In addition, the CD-CA suspension was also successfully applied to the determination of Fe^3+^ in real lake water (Supplementary Fig. 5f).

### Universality confirmation

**Fig. 5:**
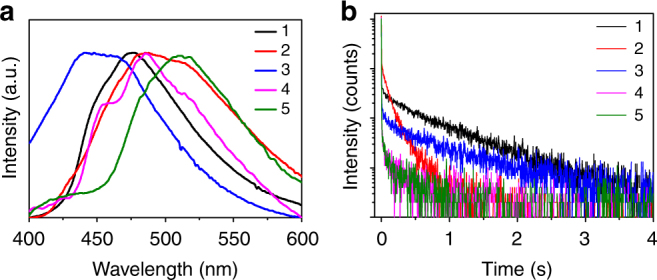
Photoluminescence properties of a series of materials in an aqueous environment. **a** Normalised phosphorescence intensity of a series of phosphorescent materials in an aqueous environment. **b** Lifetime decay profiles of a series of phosphorescent materials in an aqueous environment

To confirm the universality of our technology, we designed a series of phosphorescent materials containing a variety of host-guest materials. The materials a-CD^31^, b-CD^35^ and c-CD^32^ have been reported in previous work, and a full description of their syntheses is described in the Supplementary Information. As anticipated, the prepared materials all exhibited long phosphorescence lifetimes in solution (Fig. 5a, b, Supplementary Table 4 and Supplementary Note 1), indicating that our method can be generally applied to the establishment of RTP in metal-free organic materials in aqueous environments.

## Discussion

In summary, we have developed an efficient metal-free RTP material system in aqueous media by introducing multiple rationally designed hydrogen-bonding interactions between the CDs, CA and water molecules. The CA particle surfaces contain a large number of polar groups; thus, CA particles adsorb a layer of highly ordered water molecules (non-freezing bound water) through strong hydrogen-bonding interactions. In the presence of CDs, the particles can form robust hydrogen-bonded networks, which not only effectively rigidifies the C=O bonds of the CDs but also greatly enhances the rigidity of the entire system, leading to an enhancement of the phosphorescence. The CD-CA suspension exhibited a high phosphorescence lifetime of 687 ms under 373 nm excitation and was successfully applied in ion detection based on its visible phosphorescence. Phosphorescence detection more effectively eliminates the fluorescence background and scattered light than the fluorescence method. Therefore, our CD-CA system shows great potential for the detection of Fe^3+^ in real lake water and complicated protein solutions. We believe that the demonstrated strategy based on bound water for enabling effective RTP in aqueous media can be utilised for the development of novel metal-free organic phosphorescence materials for chemical sensors as well as for bioimaging applications in real water environments.

## Methods

### Chemicals

Unless otherwise noted, all chemicals were commercially available and used as received without additional purification. Anhydrous citric acid, urea, CA, folic acid, ethylenediamine triacetic acid (EDTA)-2Na, biuret and the oxidised form of β-nicotinamide-adenine dinucleotide were purchased from J&K Scientific Ltd (Beijing) or Aladdin Chemicals Ltd (Shanghai). Deionised (DI) water (18.2 MΩ.cm at 25 °C) prepared by a Milli-Q water system was used throughout all experiments. Lake water samples were obtained from the Weiminghu Lake of Peking University, China. Before evaluation, the lake water samples were filtered through a 0.20 µm membrane and then centrifuged at 10,000 rpm for 10 min. The resultant water samples were spiked with Fe^3+^ at different concentrations and then analysed with the proposed method.

### Material characterisation

TEM images were taken by an H-7650 instrument (Hitachi, Japan). HRTEM images were obtained on a JEM2010 high-resolution field-emission transmission electron microscope at 200 KV (JEOL, Japan). XPS was performed using an Escalab 250 XI system (Thermo Electron Corporation, USA). FTIR spectra were recorded on a Bruker Tensor 27 spectrophotometer (Germany). The absorbance spectra were recorded with a Shimadzu UV-3600 spectrometer. Steady-state PL and phosphorescence spectra were collected on a Hitachi F-7000 fluorescence spectrophotometer. Time-resolved luminescence spectra were obtained using an Edinburgh FLS920 fluorescence spectrophotometer. Lifetime decay profiles were measured using an Edinburgh FLS920 fluorescence spectrophotometer equipped with a xenon arc lamp (Xe900), a nanosecond hydrogen flash-lamp (nF920) and a microsecond flash-lamp (µF900). For the temperature-dependent experiment, the sample was placed in an Optistat DN-V liquid nitrogen cryostat with a temperature control range between 80 and 293 K. The lifetimes (*τ*) of the luminescence were obtained by fitting the decay curve with the following multi-exponential decay function:

1 $$I\left( t \right) = \mathop {\sum }\limits_i A_i\mathrm{e}^{ - \frac{t}{{\tau _i}}}$$

where *A*_*i*_ and *τ*_*i*_ represent the amplitude and lifetime, respectively, of the individual components of the multi-exponential decay profiles. DSC was performed on a DSC Q-2000 from TA Instruments using cooling and heating rates of 5 K min^−1^. DLS was performed on a Malvern Instruments Zeta Sizer Nano-ZS apparatus. ITC was performed on the Micro Cal ITC-200. Fluorescence images were taken by a CLSM equipped with a diode laser at 405 nm. The binding affinity study was investigated with an MST instrument. Raman spectra were measured on a Horiba-HR-800 Raman spectroscopy system. This system was equipped with a 514 nm diode laser source. NMR spectra were collected with a Bruker Avance Ш HD 500 MHz instrument. XRD patterns were obtained by a Rigaku S2 diffractometer.

### Synthesis of CDs

Anhydrous citric acid (1 g) and urea (0.5 g) were dissolved in DI water (25 mL) and stirred to form a clear solution. Then, the solution was transferred to a poly (tetrafluoroethylene) (Teflon)-lined autoclave (50 mL) and heated at 250 °C for 10 h. The obtained dark brown solution was centrifuged at high speed (10,000 rad min^−1^) for 20 min to remove large or agglomerated particles. The final products were obtained via freeze-drying. A stock solution (4 mg ml^−1^) of the obtained CDs was then prepared for further use.

### Preparation of CD-CA powders

Different masses of the CDs (0.2 mg, 0.4 mg, 0.8 mg, 2 mg, 4 mg, and 16 mg) were first diluted with 4 mL of DI water and mixed with CA (0.75 g) under constant stirring for 12 h. The reaction mixtures were then centrifuged (8000 rpm, 10 min) to remove the unabsorbed CDs, and the solid powders remaining on the bottoms of the centrifuge tubes were fully dried under vacuum. Finally, the CD-CA powders were obtained and then redissolved in water for further use. Unless stated otherwise, the CD-CA powder in this article was prepared by mixing CDs (2 mg) and CA (0.75 g) in 4 mL of DI water.

### Synthesis of a-CDs

The a-CDs were prepared according to previous work. EDTA-2Na (1.6 g) was placed in a quartz boat and heated in a tube furnace under a N_2_ atmosphere at a rate of 10 °C min^−1^ to 400 °C. After annealing for 4 h at 400 °C, the reaction was allowed to cool to room temperature, and the black product was dispersed in DI water (80 mL) under ultrasonication for 30 min. Then, the solution was centrifuged at 9500 rpm for 16 min to remove larger and insoluble particles. Solid-state luminescent CDs were obtained via freeze-drying.

### Synthesis of b-CDs

The b-CDs were prepared according to previous work. In brief, folic acid (FA) (1 g) was dissolved in DI water (100 mL). Then, the solution was transferred to a Teflon-lined autoclave (200 mL) and heated at 260 °C for 2 h. After the reaction, the reactor was cooled to room temperature naturally. The final product was collected following centrifugation at 10,000 rpm for 20 min.

### Synthesis of c-CDs

The c-CDs were prepared according to previous work. Isophorone diisocyanate (8 g, 36 mmol) was added to a Teflon vessel that was sealed with an explosion-proof enclosure. Then, raw N-doped carbon quantum dots (CQDs) were prepared under 700 W microwave irradiation at 250 °C for 10 min. The oil-soluble CQDs were collected after centrifugation and dialysed. Then, the oil-soluble CQDs were ultrasonically dispersed in DI water (50 mL) and heated at 180 °C for 3 h. After the reaction, the mixture was cooled to room temperature, and the obtained yellow solution was centrifuged (10,000 rpm, 20 min) and filtered through a 0.22 mm filter membrane to remove large and insoluble particles. Solid-state luminescent a-CDs were obtained via freeze-drying.

### Fluorescent detection of Fe^3+^ ions

The detection of Fe^3+^ was performed in an ultrapure water solution. An aqueous solution of CDs (0.01 mg mL^−1^) was used as the blank solution. Different concentrations of Fe^3+^ were added to the CD blank solution, and the mixed solutions were equilibrated for 10 min. In addition, the PL emission spectra were collected at an excitation wavelength of 373 nm. The selectivity for Fe^3+^ was confirmed by adding other metal ion stock solutions instead of Fe^3+^ in a similar way. All experiments were performed at room temperature.

### Phosphorescent detection of Fe^3+^ ions

The detection of Fe^3+^ was performed in an ultrapure water solution. Different concentrations of Fe^3+^ were added to 3 mL of the CD solution (0.01 mg mL^−1^), and the mixed solutions were equilibrated for 10 min. Then, the same amount of CA powder (0.3 g) was added to each mixed solution, which was stirred for 1 h. Then, the PL emission spectra were collected at an excitation wavelength of 373 nm. The selectivity for Fe^3+^ was confirmed by adding other metal ion stock solutions instead of Fe^3+^ in a similar way. All experiments were performed at room temperature. The detection of Fe^3+^ in lake water and complicated protein solutions was carried out using lake water and complicated protein solutions, respectively, instead of ultrapure water. The complicated protein solution was prepared by adding peptone (0.5 g) to ultrapure water (50 mL).

### Data availability

The data that support the findings of this study are available from the corresponding author.

## Electronic supplementary material

Supplementary Information
