# Supplementary material for: Induction of long-lived room temperature phosphorescence of carbon dots by water in hydrogen-bonded matrices
Source: Nat Commun. 2018 Feb 21;9:734. doi: 10.1038/s41467-018-03144-9 (PMC5821822; doi:10.1038/s41467-018-03144-9)
Supplement: Supplementary file 1 — Supplementary Information [file 41467_2018_3144_MOESM1_ESM.pdf]

Induction of long-lived room temperature phosphorescence of carbon dots by water in hydrogen-bonded matrices

Li et al

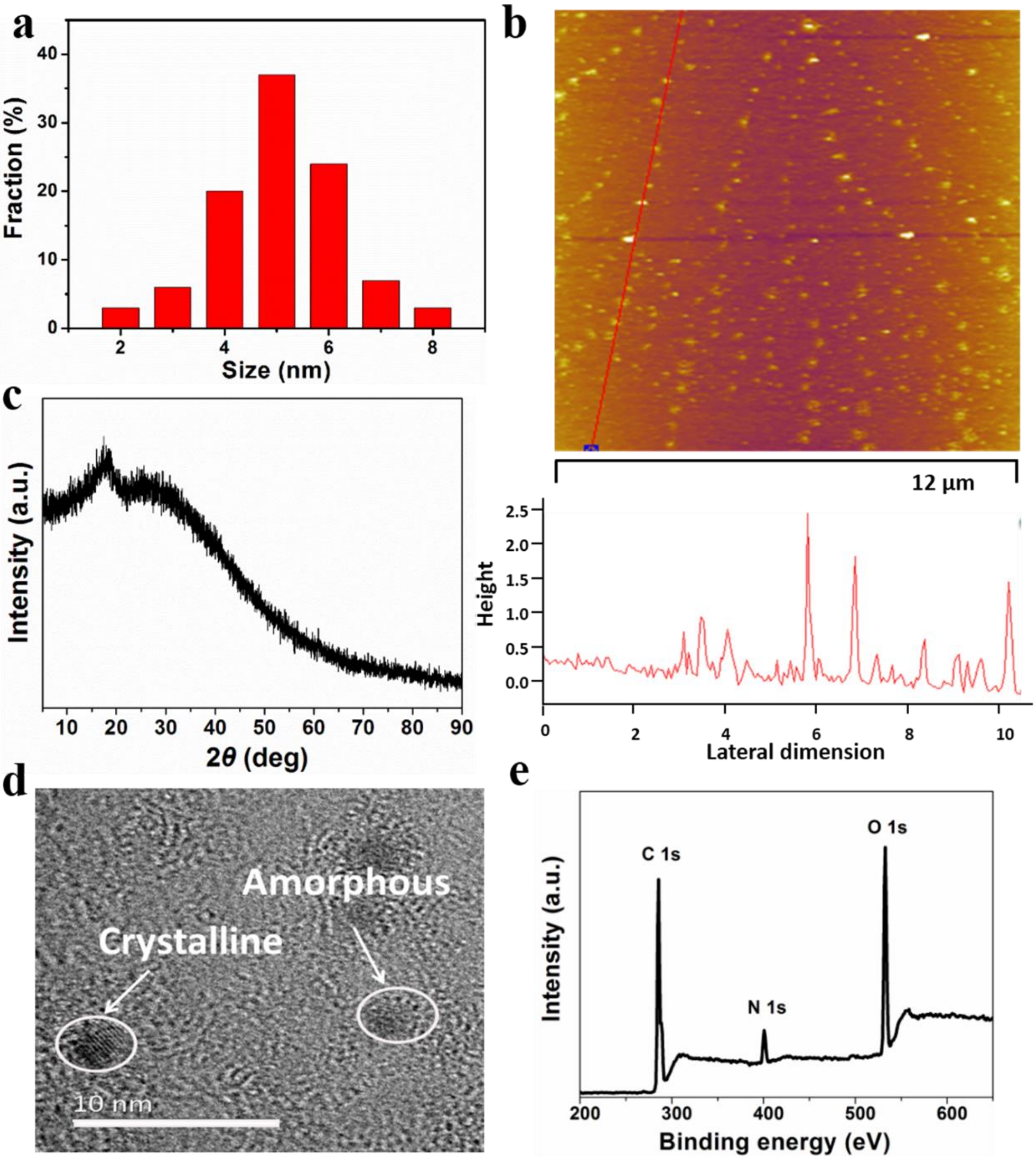

Supplementary Figure 1. a) Size distribution of CDs. b) AFM image and size distributions of CDs. Height profiles are given for the marked red line in the AFM image. c) XRD spectrum of CDs. d) HRTEM image of the CDs. e) XPS spectrum of CDs.

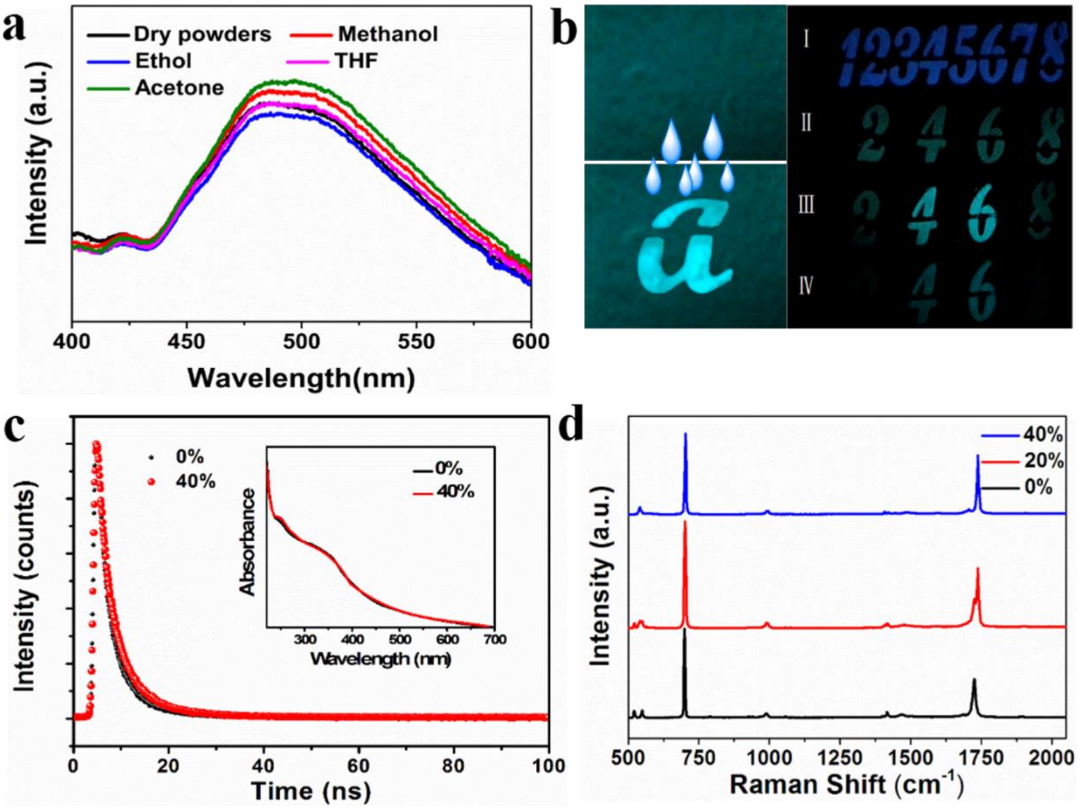

Supplementary Figure 2. a) Phosphorescence spectra of CDs-CA system with various solvents excited at 373 nm, respectively. b) A mask image of "a" on dry CD-CA powder before and after adding water under UV excitation (left); Security protection applications (right). c) Fluorescence decay profiles of CD-CA with 0% and 40% water contents, respectively; Inset is corresponding absorption spectra. d) Raman spectra of CA with 0%, 20% and 40% water contents, respectively.

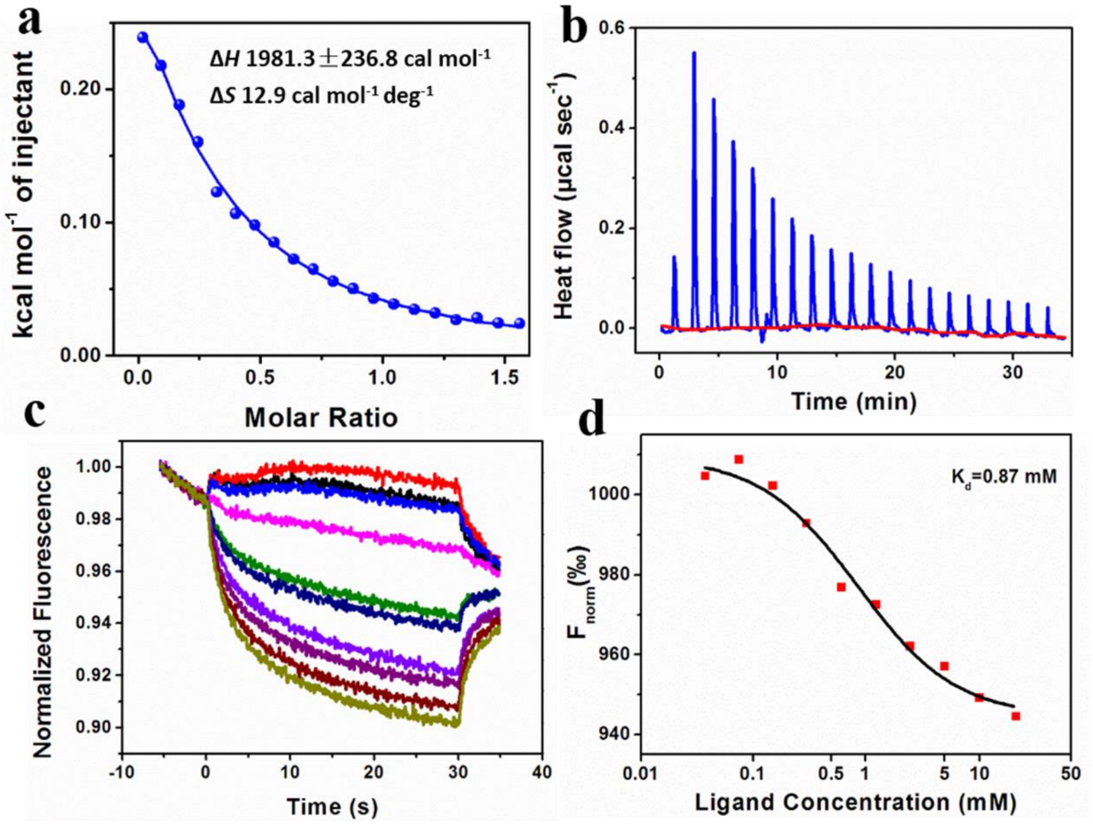

Supplementary Figure 3. a) Non-linear fitting curve of the binding heat versus the molar ration of CDs to CA. b) ITC profile for the binding of CDs (100 μg ml^{-1}) to CA (20 mM). c) Binding curves of CDs and CA obtained from MST fixed with 80% LED power. The CDs concentration kept constant at 50 μg/ml. Concentration of CA was ranging from 20 mM to 3.75 μM. d) Plotting of the change in thermophoresis and concomitant fitting of the data yielded a K_d of 0.87 ± 0.28 mM.

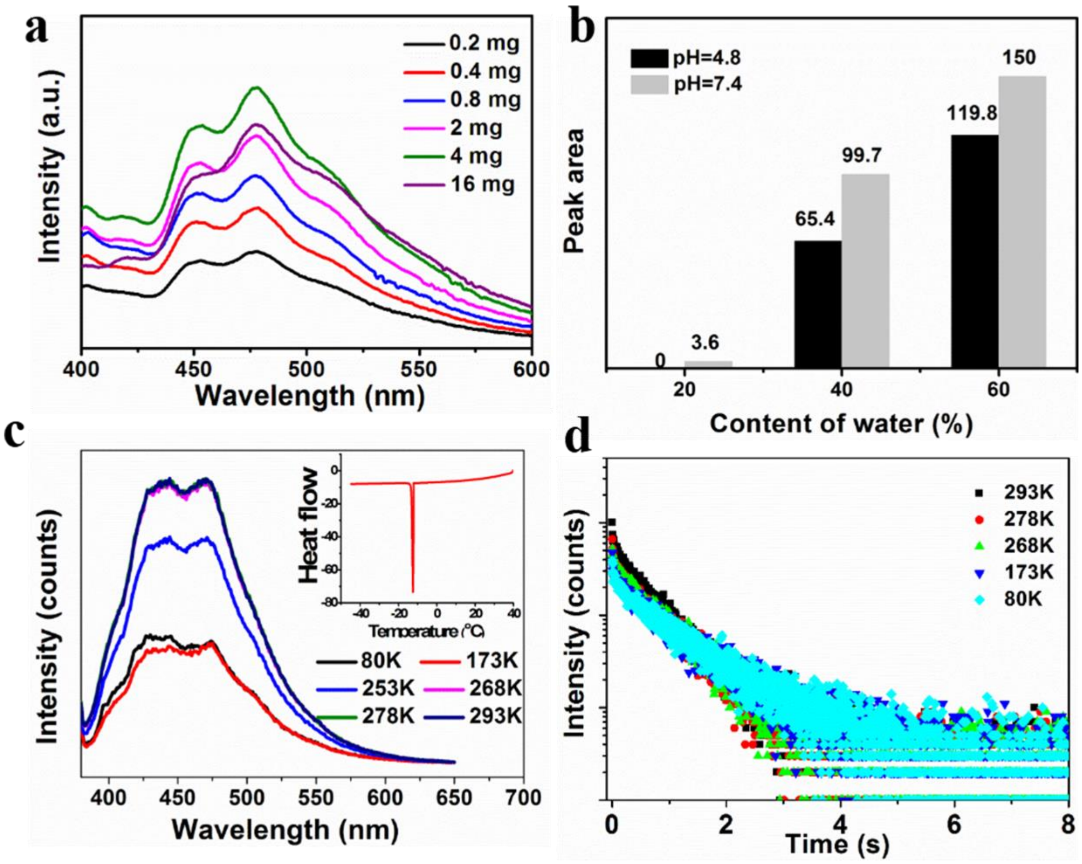

Supplementary Figure 4. a) Phosphorescence spectra of CD-CA system at 40% water content with different CDs contents excited at 373 nm, respectively. b) DSC endothermic area of CD-CA system with different water contents at pH 4.8 and 7.4, respectively. c) Phosphorescence spectra of CD-CA system with water content 40% excited at 373 nm at temperatures from 80-293 K. Inset is the corresponding DSC cooling curve at temperatures from 40 ^{o}C to -40 ^{o}C; The sharp peaks at -14 ^{o}C correspond to the water freeze. d) Lifetime decay profiles of CD-CA system with water content 40% excited at 373 nm at temperatures from 80-293 K.

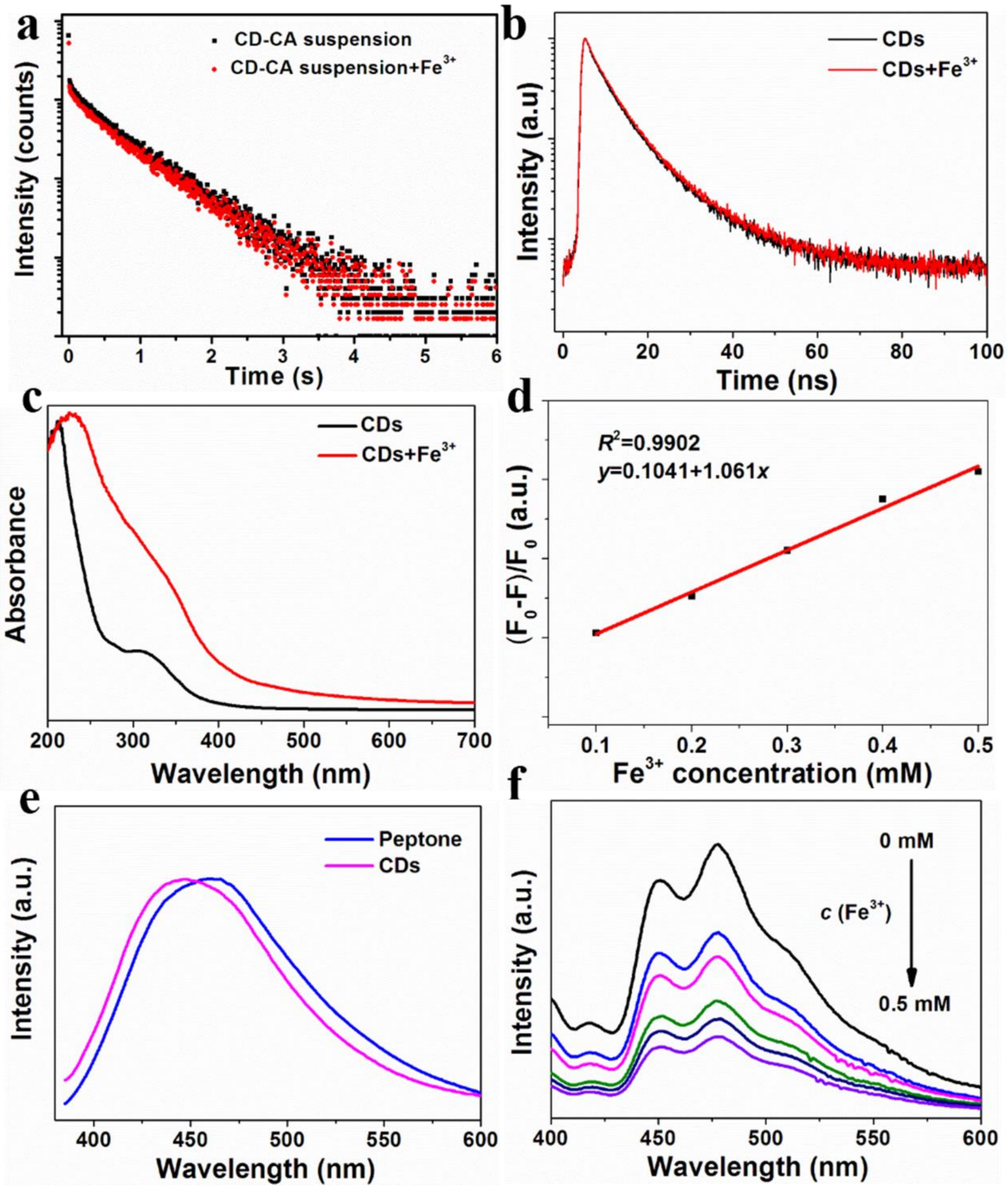

Supplementary Figure 5. a) Phosphorescence decay profiles of CD-CA suspension alone and CD-CA suspension+Fe^{3+}. b) Fluorescence decay profiles of CDs alone and CDs + Fe^{3+}. c) Absorption spectra of CDs alone and CDs + Fe^{3+}. d) Stern–Volmer plot as a function of Fe^{3+} concentration in peptone solution. e) Fluorescence spectra of CDs and peptone excited at 373 nm, respectively. f) Phosphorescence spectra of CDs-CA suspension in lake at various calculated concentrations of Fe^{3+} 0, 0.1, 0.2, 0.3, 0.4 and 0.5 mM from top to bottom.

Supplementary Table 1. Zeta potentials of CDs and CA at different pH values.

pH	2.6	3.1	4.8	7.4	8.7	10.1
CDs	2.89 mV	2.37 mV	-5.14 mV	-25.2 mV	-28.1 mV	-34.9 mV
CA	6.76 mV	6.84 mV	-1.26 mV	-10.5 mV	-22.1 mV	-30.8 mV
△[a]	9.65 mV	9.21 mV	6.4 mV	35.7 mV	50.2 mV	65.7 mV

Δ [a] stand for the sum of the absolute values of zeta potentials of CDs and CA

Supplementary Table 2. Photoluminescence lifetimes (τ) of CD-CA system at 40% water content with different CDs contents under ambient conditions

Content (mg)	τ_1 (ms)	A_1 (%)	τ_2 (ms)	A_2 (%)	τ_{ave} (ms)	Total QY (%)
0.2	53.23	8.07	669.3	91.93	665	18.8
0.4	63.3	7.23	686.2	92.77	682	17.6
0.8	50	5.31	681.3	94.69	678	16.5
2	55.23	5.71	690.3	93.4	687	15.6
4	50	7.23	664.7	92.77	661	12.3
16	50	7.82	652.8	92.18	648	6.2

Note: the excitation wavelength was 373 nm, and lifetime was monitored by the emission wavelength at 480 nm.

Supplementary Table 3. Photoluminescence lifetimes (τ) of CD-CA system with 40% water content at different temperatures from 80 K to 293 K

Temperature (K)	τ_1 (ms)	A_1 (%)	τ_2 (ms)	A_2 (%)	τ_{ave} (ms)
80	150.8	7.35	1045	92.45	1034.8
173	73.33	4.33	944.5	95.67	941.4
253	80.21	5.71	886.3	94.29	881.9
268	92.59	5.52	768.6	94.48	763.9
278	109.40	8.43	754.1	91.57	745.6
293	161.11	10.25	701.1	89.75	687.2

Note: the excitation wavelength was 373 nm, and lifetime was monitored by the emission wavelength at 480 nm.

Supplementary Table 4. Photoluminescence lifetimes (τ) of a series of materials in water environment.

	Gest	Host	τ_1 (ms)	A_1 (%)	τ_2 (ms)	A_2 (%)	τ_3 (ms)	A_3 (%)	τ_{ave} (ms)
1	a-CDs[1]	CA	21.55	1.74	198	11.08	814.8	87.18	795.92
2	b-CDs[2]	CA	4.66	1.78	71.98	62.97	230.5	35.25	173.6
3	c-CDs[3]	CA	6.84	2.24	118.7	9.88	941.9	87.87	930.2
4	Our CDs	Biuret	1.215	23.62	32.22	21.68	306.9	54.70	295.4
5	NAD	CA	11.12	27.08	159.0	72.92	/	/	155.3

Supplementary Note 1: Characterisation of the CD-CA powder

In Supplementary Figure 2b, the patterned security feature of '2468' was filled with mixture of phosphorescent CD-CA powders and fluorescent powders, while '1357' was only filled with fluorescent powders. The '2468' was encrypted in '12345678' under the excitation of 365 nm UV-light owing to fluorescence background of fluorescent powders (Ⅰ). However, when the UV-light is turned off, the encryption of '2468' could be readily visualized (Ⅱ). After water-spraying treatment '46', water greatly enhanced phosphorescent lifetime and intensity (Ⅲ). After a period of time, only '46' could be observed (Ⅳ). Therefore, our phosphorescent materials could be used to double anti-counterfeiting field. In Supplementary Figure 2d, typical Raman peaks of solid dry state CA at 523, 702, and 1725 cm-1 were observed. The peak at 525 cm^{-1} is assigned to ring out-of-plane bending. The peaks at 702 and 1725 cm^{-1} arise from C=O in-plane bending and C=O stretching, respectively. As the contents of water increased from 0% to 40%, the peak at 1725 cm^{-1} becomes weaker and then red shifts to 1738 cm^{-1}, meanwhile the peak at 523 cm^{-1} red shifts to 541 cm^{-1}. These results clearly indicate that C=O on CA surface have a strong hydrogen bond interaction with water molecules. In Supplementary Figure 3b, values of entropy and enthalpy don't reflect real binding thermodynamic properties of CDs and CA because of the unknown CDs concentration, but observed endothermic signal and positive values of ΔS and $T^*\Delta S > \Delta H$ are reliable. According to these results, we can conclude that the binding of CDs with CA molecule is partly driven by hydrophobic interaction. In Supplementary Figure 4b, robust exothermic signals were observed at water content 20%, 40%, and 60%, respectively, overall more exothermic heats than that at pH 4.8, which means a corresponding bound water content decrease in CD-CA system at pH 7.4. The multiple phosphorescent lifetimes (Supplementary Table 2-4) imply various electronic transition processes, which may be due to a wide range of chemical environments on the surface of CDs. The average lifetimes were calculated using the equation,

$$\langle\tau\rangle = \sum\alpha_i \tau_i^2 / \sum\alpha_i\tau_i, \quad (1)$$

where A_i and τ_i represent the amplitudes and lifetimes, respectively, of the individual components for multi-exponential decay profiles.

Supplementary References

1. Deng, Y. *et al*. Long lifetime pure organic phosphorescence based on water soluble carbon dots. *Chem. Commun*. **49,** 5751-5753 (2013).

2. Li, Q. *et al*. Efficient room-temperature phosphorescence from nitrogen-doped carbon dots in composite matrices. *Chem. Mater*. **28,** 8221-8227 (2016).

3. Tan, J., Zhang, J., Li, W., Zhang, L. & Yue, D. Synthesis of amphiphilic carbon quantum dots with phosphorescence properties and their multifunctional applications. *J. Mater. Chem. C* **4,** 10146-10153 (2016).
